# Developing inclusive digital health diagnostic for schistosomiasis: a need for guidance via target product profiles

**DOI:** 10.3389/fpara.2023.1255848

**Published:** 2023-09-26

**Authors:** Adeola Onasanya, Michel Bengtson, Ludo de Goeje, Jo van Engelen, Jan-Carel Diehl, Lisette van Lieshout

**Affiliations:** ^1^ Department of Sustainable Design Engineering, Faculty of Industrial Design Engineering, Delft University of Technology, Delft, Netherlands; ^2^ Department of Parasitology, Leiden University Medical Center, Leiden, Netherlands

**Keywords:** schistosomiasis, Target Product Profile, digital diagnostics, inclusiveness, design

## Abstract

**Introduction:**

The INSPIRED project aims to develop inclusive Digital Optical Diagnostic Devices (DODDs) for schistosomiasis, to support disease management by enabling rapid diagnostic results, to improve efficient data management to guide decision-making and to provide healthcare workers with critical health information to facilitate follow-up action. Due to the non-availability of Target Product Profiles (TPPs) for guiding the development of digital diagnostics for schistosomiasis, we explored existing diagnostic TPPs.

**Methods:**

Using a curated open access database (Notion database), we studied a selection of TPPs for diagnosing infectious diseases, focusing on specifications related to digital health products for Neglected Tropical Diseases (NTDs).

**Results:**

Eighteen TPPs originating from 12 documents, covering 13 specific diseases, were selected and their characteristics were labeled and entered into the database. Further exploration of the database revealed several gaps, including a lack of stakeholder input, sustainability, and TPP availability. Other significant gaps related to digital health platform interconnectivity and data stewardship specifically in relation to digital diagnostics, including DODDs.

**Discussion:**

These findings reflect two possible scenarios: (1) there is currently no need for digital diagnostic devices for schistosomiasis and, by extension for other NTDs; or (2) those needs are not yet covered by TPPs. Therefore, we recommend that digital health diagnostics are included in the use cases for schistosomiasis control and elimination, at least in the ideal/desirable scenario, as this will guide research and incentivize investment in digital health diagnostics for schistosomiasis.

## 1 Introduction

Schistosomiasis is a Neglected Tropical Disease (NTD) prevalent in rural communities in tropical regions and affecting more than 250 million people worldwide (World Health Organization, 2023b). The WHO’s NTD 2021-2030 roadmap sets global targets and milestones to prevent, control, and eliminate diseases and one of the action points is the development of new tools and diagnostics (World Health Organization, 2020a).

In 2021 two use cases for schistosomiasis diagnostics became available, one at the individual patient level (test and treat) and the second for diagnosis at the population-level (monitoring and evaluation) (WHO, 2021). Considering the individual level, accurate diagnostics are mostly needed at the primary health care level, where patients seek medical attention. On the other hand, population-level-based diagnostic strategies involve screening of whole communities or school-age children, and also includes sample pooling procedures. In both situations, Point-of-Care (POC) diagnostic approaches play an essential role (Cavalcanti et al., 2019; Hoekstra et al., 2021).

Currently, diagnostic tests for schistosomiasis include egg detection by microscopy, the measurement of morbidity markers such as haematuria, Nucleic Acid Amplification Tests (NAAT) and immunological tests, such as antibody detection and antigen detection tests, including the urine-based lateral flow test for the detection of Circulating Anodic Antigen (POC-CCA) (Ajibola et al., 2018; Casacuberta-Partal et al., 2019; Hoekstra et al., 2021). Newer digital diagnostic tests are currently undergoing development, focusing on improving the ease of use of current optical and immuno-chemistry-based tests by reducing potential human error and diminishing ergonomic problems (Davies et al., 2017; Privitera et al., 2017; Vojnov et al., 2019; Mewamba et al., 2021). Some of these devices are being implemented for use in NTDs (Mewamba et al., 2021), haematology (Bachar et al., 2021) and cancer diagnostics (Gola & Doyle-Lindrud, 2016; Valvert et al., 2021). Digital technology-based tests can also be implemented with fewer and less technically trained staff within the limitations posed by the current human resource health challenges in Africa (Petti et al., 2006; Davies et al., 2017).

The INSPiRED consortium (http://inspired-diagnostics.info/) is developing Digital Optical Diagnostic Devices (DODDs; **Table 1**) for schistosomiasis, which include automated digital microscopes and optical readers for lateral flow tests (Meulah et al., 2022b; Oyibo et al., 2022). Other groups are also developing optical digital devices for the detection of helminth eggs in urine or stool samples or the reading of the POC-CCA urine strip test (Bogoch et al., 2014; Holmström et al., 2017; Mewamba et al., 2021; Armstrong et al., 2022; Ward et al., 2022). In parallel, there are initiatives for NAAT-based diagnostics coupled with digital technology which could potentially be applied to NTDs (Cunnington and The Digital Diagnostics for Africa Network, 2022). These digital diagnostic devices are part of a wide range of Digital Health Products (DHPs) that are being explored to support traditional methods of diagnosis, treatment, monitoring and evaluation of diseases.

**Table 1:** Definition of key terms.

Terms	Definition
Digital Health Products (DHPs)	ICT-based health technologies including software (databases, platforms, web applications) and/or hardware (devices) with/without interconnectivity.
Digital Health Diagnostics (DHDs)	Digital health products that use digital data to make or support disease diagnosis.
Digital Optical Diagnostic Devices (DODDs)	Hardware (devices) using optical systems (automated/semi-automated) and digital data to make or support disease diagnosis.

Digital Health Diagnostics (DHDs) are DHPs using digital data to make or support disease diagnosis. They can be used by patients, health workers and health policymakers to diagnose and manage diseases and health risks, promote wellness, and support health-related decision-making processes (Ronquillo et al., 2022). They have the potential to reduce inefficiencies related to manual procedures, thus improving access and quality of care, and increasing ownership of health by patients through personalized care (Center for Devices and Radiological Health, 2020).

As developers, we envision that DODDs, a subset of DHDs, could play an important role in controlling and eliminating schistosomiasis in low and middle-income countries (population-level use case). The digital capture of optical images from DODDs can substantially improve data quality and aid prompt decision-making by providing digital data to support faster diagnostic decision-making. Notably, our recent literature review demonstrated that most DODDs for schistosomiasis are limited to the proof of concept stage, and almost no devices are implemented and/or commercially available (Meulah et al., 2022a).

Guidance for developers of diagnostic tests, including optimal and minimal parameter specifications, typically in the form of use cases combined with Target Product Profiles (TPPs) endorsed by organizations like the WHO and/or other international organizations, is a key factor to entice product developers, especially in areas like NTDs where the economic attractiveness is limited. TPPs are intended to guide researchers, policymakers, commercial parties, and funders toward the development of health products that fit the particular needs of a given context.

**Figure 1:**
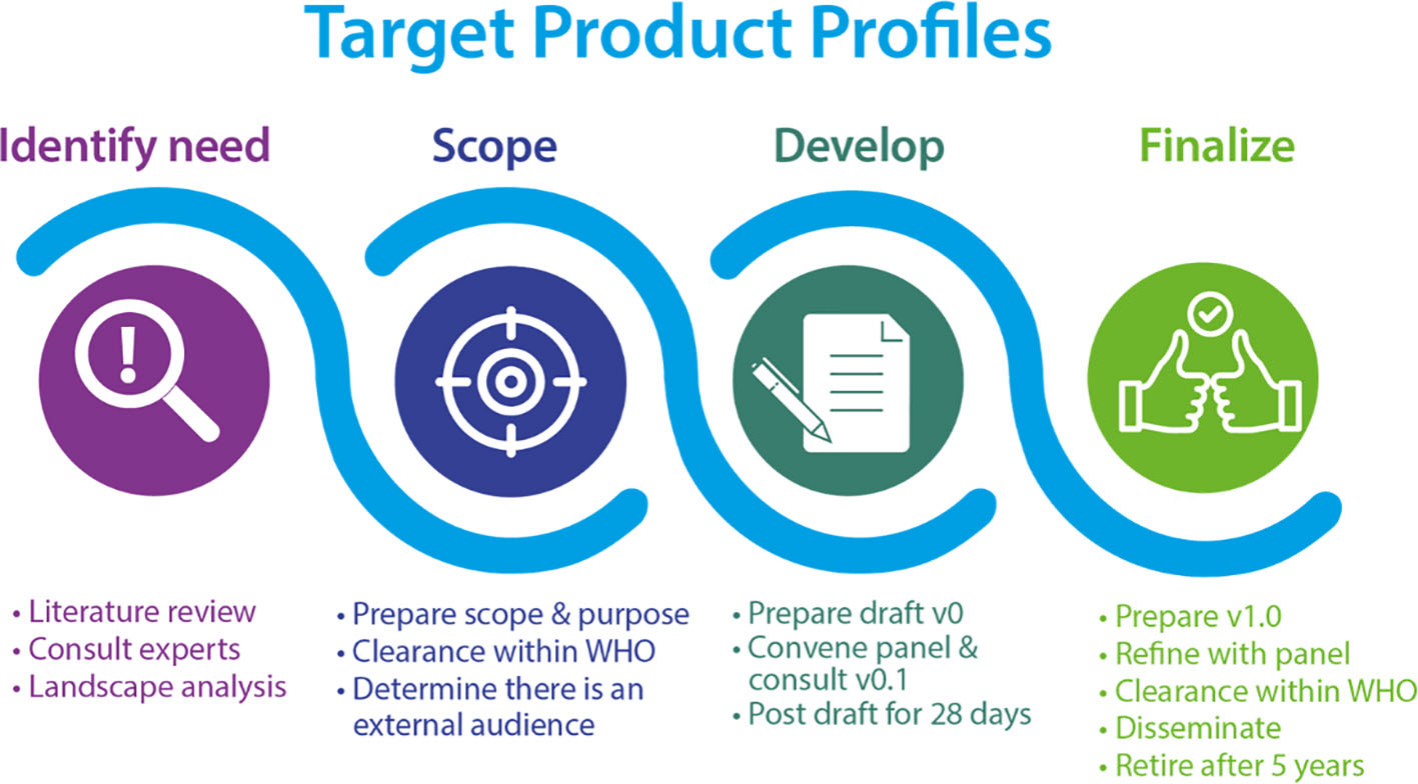
The WHO TPP drafting process (World Health Organization, 2023a) CC BY-NC-SA 3.0 IGO.

The Diagnostic Technical Advisory Group (DTAG), commissioned by WHO, is the working group with a coordinating role in the development of TPPs (**Figure 1**) for NTDs to reach the goals described in the 2020-2030 roadmap (World Health Organization, 2020b). The diagnostic TPPs for schistosomiasis, developed by DTAG, are for (1) monitoring and evaluation of control programmes and (2) determining if the transmission has been sufficiently interrupted and when to conduct post-mass drug administration (MDA) surveillance (World Health Organization, 2020b).

Although developing DHDs for schistosomiasis, and by extension, other NTDs, will make a substantial contribution to meeting WHO targets, there are currently no guidelines to support developers in this process. A broad search for TPPs of DHDs for schistosomiasis and other NTDs yielded hardly any information. The WHO Health Product Profile Directory (HPPD), which is a database of currently available health products targeting low-resource settings, includes ‘digital health’ as a product type. However, a search of the directory on June 15, 2023, revealed that DHPs are only available for TB and clinical decision support mainly focused on disease management and awareness. Furthermore, while many TPPs exist in the database, only a few of those TPPs are dedicated to digital products in the context of global health or at least provide some guidance on digital connectivity.

For schistosomiasis specifically and other NTDs broadly, there are no existing TPPs published by health organizations including the WHO that describe the needs for and characteristics of DHPs to facilitate schistosomiasis diagnostics development. This could either indicate there is no perceived need for DHPs and by extension DHDs and DODDs in this context, or it could mean those needs are not specified and described yet. According to the road map for neglected tropical diseases 2021-2030 (World Health Organization, 2020a), digital health platforms for collecting and monitoring data are needed. However, this need is not further specified. While the WHO, has made an effort to include ‘digital health’ as part of the need for NTD elimination, it is still unclear how and what this need should entail.

This paper discusses the need for DHDs for schistosomiasis elimination and highlights the gaps in the current TPPs for schistosomiasis, particularly the absence of guidance for the development of digital diagnostics that can support the elimination of schistosomiasis. The paper addresses ways to improve TPP inclusivity, taking into consideration the importance of specific digital health concepts that do not apply to currently available TPPs.

## 2 Methodology

To explore what we can learn from existing TPPs concerning the need for DHDs for schistosomiasis, a publicly accessible database consisting of a selection of TPPs was curated (Notion database). Documents for the database were obtained via a general web search using the Google search engine. The Google search was limited to the first six pages of the database. Other websites and databases of NGOs searched include PATH, FIND, MSF and WHO. The search was performed in June 2022 and only non-profit health organizations such as the WHO were included, in order to reduce the potential risk of conflict of interest by TPPs constructed by private-for-profit companies.

The inclusion criteria for a TPP to be entered into the notion database were based on the year of publication (≥ 2016) and three domains: (1) diagnostic tests using human samples to support mass drug administration programs (MDA) for NTDs; (2) diagnostic tools and digital devices to aid the detection of NTDs, HIV/AIDS, TB and malaria; (3) diagnostic systems such as digital health applications for NTDs, HIV/AIDS, TB and malaria. While there are 21 NTDs, only NTDs that include MDA as a treatment approach were included in the database (World Health Organization, 2020a). HIV/AIDS, TB and malaria were selected based on their global health relevance. Based on this selection criteria, the TPPs for the following NTDs were then included in the database: foodborne trematodiases; lymphatic filariasis; onchocerciasis; scabies and other ectoparasites; schistosomiasis; soil-transmitted helminthiases; taeniasis/cysticercosis; and yaws and other endemic trepanemotoses.

Thereafter, the following search terms were used to find relevant TPPs for analysis in the notion database:

Target Product Profile + Lymphatic filariasis, onchocerciasis, river blindness, schistosomiasis, ascariasis, trichuriasis, hookworm, soil-transmitted helminthiases, trachoma, foodborne trematodiases, taeniasis, cysticercosis, yaws, scabies.

Target Product Profile + Digital device, electronic device, digital health, e-health, smart device.

Target Product profiles + WHO, FIND, GHIT, PATH, Bill & Melinda Gates Foundation, UNICEF.

The contents of each TPP were categorized in the database according to predefined characteristics that are standardized in formal TPPs (i.e., all minimum and ideal characteristics were labeled in the database within a category). In this way, the contents of different TPPs can be easily searched and compared, and metadata can be analyzed. For easy handling, sharing and the functionality of adding and filtering multiple labels, the web-based application Notion was used to analyse the database.

Metadata was added to single characteristics and requirements as stated in the TPP, in the form of labels (e.g. context and user; functionality; performance; market and business). This database allows the user to categorize TPPs based on the specified use case (i.e., individual case management; mapping; monitoring and evaluation; stopping decisions; surveillance).

## 3 Results

**Figure 2:**
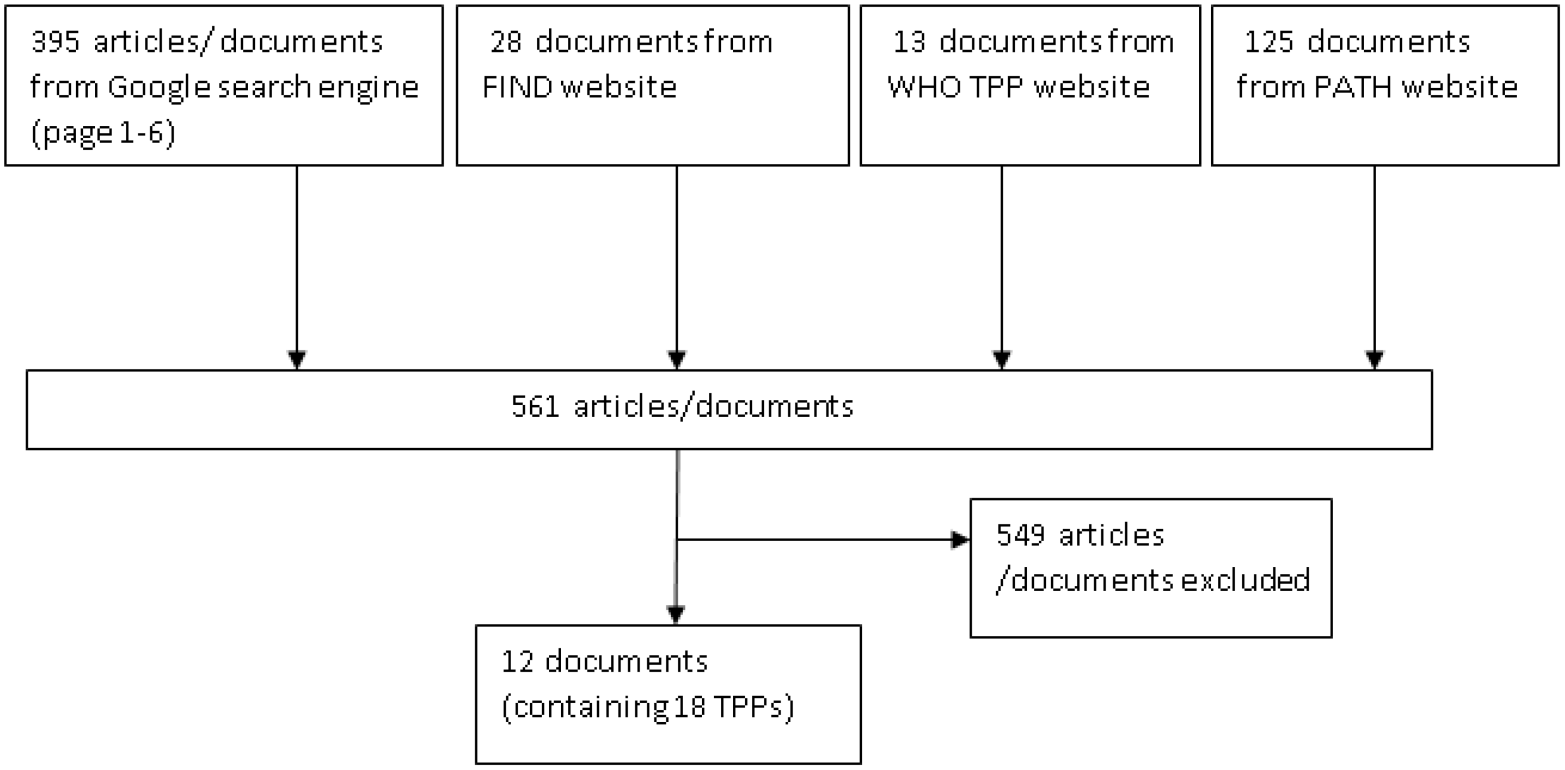
Flow chart of search method.

**Figure 2** demonstrates the process of data collection for the Notion database which is publicly available and can be explored by users. 18 TPPs that matched the criteria were found. The 18 TPPs originated from 12 documents. Some documents contained more than one TPP, for example, different TPPs for different use scenarios. The WHO was the (co)initiator of 15 of the 18 TPPs and 12 TPPs were published between 2020 to 2022. 14 TPPs were related to the first search terms while the other 4 TPPs were related to digital devices and digital health (search terms 2 & 3), as found in **Table 2** and the appendix. Two of those four TPPs target mobile phone use, while the other two describe dedicated digital devices. 12 TPPs mentioned stakeholders involved in the drafting process with a minimum of 6 to a maximum of 52 stakeholders. Only 2 TPP documents had TPP meeting notes available. 8 TPP development processes were sponsored by a funding agency, foundation or fund.

**Table 2:** Overview of database information.

Initiator	Year	Target Product Profile	Targeted disease(s)	Disease control stage
WHO	2021	Diagnostic target product profiles for monitoring and evaluation of schistosomiasis	Schistosomiasis	Monitoring and evaluation
WHO	2021	2 Diagnostic target product profiles for transmission interruption and surveillance	Schistosomiasis	Stopping decisions, surveillance
WHO	2021	Diagnostic test for surveillance of lymphatic filariasis: Target product profile	Lymphatic filariasis	Surveillance
WHO	2021	Diagnostic test for lymphatic filariasis to support decisions for stopping triple therapy mass drug administration: target product profile	Lymphatic filariasis	Stopping decision
WHO	2021	Onchocerciasis: diagnostic target product profile to support preventive chemotherapy -mapping	Onchocerciasis	Mapping
WHO	2021	Onchocerciasis: diagnostic target product profile to support preventive chemotherapy - stopping decision	Onchocerciasis	Stopping decision
WHO	2021	Diagnostic target product profile for monitoring and evaluation of soil-transmitted helminth control programs	Soil-transmitted helminthiases	Monitoring and evaluation
Bill & Melinda Gates Foundation	2018	Target product profile for STH use-case #3 diagnostic (confirming decision to stop population-level intervention). STH, soil-transmitted helminth	Soil-transmitted helminthiases	Monitoring and evaluation, stopping decision
Bill & Melinda Gates Foundation	2018	Target product profile for STH use-case #1 and #2A diagnostic (mapping, monitoring population-level intervention). STH, soil-transmitted helminth	Soil-transmitted helminthiases	Mapping, monitoring and evaluation
WHO	2017	TPP for a specific test for the detection of T. solium taeniasis in humans (various platforms)	Taeniasis	Monitoring and evaluation, surveillance
WHO	2017	TPP for a point-of-care test for the detection of Taenia solium taeniasis in humans	Taeniasis	Individual case management, monitoring and evaluation, surveillance
WHO	2022	2 Target product profile for the detection of a case of yaws and the detection of azithromycin resistance	Yaws	Mapping
WHO	2022	Scabies Diagnosis TPP – Mass Drug Administration Start	Scabies	Mapping
WHO	2022	Scabies Diagnosis TPP – Mass Drug Administration Stop	Scabies	Stopping decision
FIND, WHO	2020	Electronic clinical decision support algorithms incorporating point-of-care diagnostic tests in low-resource settings: a target product profile	Diseases in low resource settings that require point-of-care diagnosis	Individual case management
WHO	2016	Target Product Profiles for digital health products for the End TB Strategy; Chapter 3: Diagnostic device connectivity for TB	Tuberculosis	Mapping, monitoring and evaluation, stopping decisions, surveillance
FIND	2020	Target Product Profile for a mobile app to read rapid diagnostic tests to strengthen infectious disease surveillance	Infectious diseases	Surveillance
WHO, FIND, MSF	2018	A Multiplex Multi-Analyte Diagnostic Platform	Severe febrile illness, HIV, Malaria, TB	Individual case management

## 4 Discussion

Based on the content of the database, two broad themes emerged in relation to the gaps and opportunities for improving current TPPs applicable to DHDs. Theme 1 focuses on inclusivity and addresses gaps like TPP availability, stakeholder inclusion, and sustainability. Theme 2 applies mainly to interconnectivity and data stewardship which are DHP-specific considerations that are applicable to both DHDs and DODDs.

### 4.1 Theme 1: inclusivity

#### 4.1.1 TPP availability

One of the barriers to DHD device development is the lack of availability and accessibility of TPPs. For example, at the time of this study, there were no TPPs for developing DHDs for schistosomiasis within the WHO health product directory. This absence can impact the DHD product development process and lead to a waste of time and financial resources in developing products which may not fit into the context of use or duplication of healthcare products. Conversely, this absence causes a lack of incentive for developers to invest in digital health products as they often rely on the TPPs before product development is initiated.

Unfortunately, the HPPD webpage was not available online during the development of our database (2021-2022), due to upgrades of the HPPD platform (personal communication with WHO representatives). In our view, this creates a lack of clarity for those who are not directly involved in the process of constructing TPPs. The transparency for end users could be improved, not only by regular updating existing TPPs, as they should be living documents, but also by maintaining a version history available online of each TPP and the HPPD webpage. Preferably TPP updates should be performed in parallel with reviewing NTD targets.

#### 4.1.2 Stakeholder inclusion

The TPP drafting process involves consultation with stakeholders. Of the 18 selected, 12 TPPs mentioned the types of stakeholders participating in the drafting process (**Table 3**). While it is sensible to determine relevant stakeholders per use-case (as TPPs are developed per use case); the extent of stakeholder involvement in the TPP development process is unclear. For instance, we found no published list of stakeholders consulted in the TPP drafting process in the documents reviewed. Only 2 TPPs had meeting notes available (these were both from the NGO database). Although the inclusion of end user involvement in the TPP drafting process is documented in the DTAG process, (WHO, 2021), we found the roles of these end users not clear. Considering and giving preference to stakeholders from schistosomiasis dominant areas is critical to improving contextual fit and uptake.

**Table 3:** TPP stakeholder information.

Initiator	Year	Disease(s)	Stakeholder type	Group size	Meeting notes available
WHO	2021	Schistosomiasis	No information	22	No
WHO	2021	Schistosomiasis	No information	22	No
WHO	2021	Lymphatic filariasis	Lymphatic filariasis technical experts, end users and other stakeholders	14	No
WHO	2021	Lymphatic filariasis	Lymphatic filariasis technical experts, end users and other stakeholders	14	No
WHO	2021	Onchocerciasis	No information		No
WHO	2021	Onchocerciasis	No information		No
WHO	2021	Soil-transmitted helminthiases	No information	14	No
Bill & Melinda Gates Foundation	2018	Soil-transmitted helminthiases	“Diverse group of key opinion leaders”	48	No
Bill & Melinda Gates Foundation	2018	Soil-transmitted helminthiases	Experts from academic institutions, industry, and private and public sectors	48	No
WHO	2017	Taeniasis	Experts from academic institutions, industry, and private and public sectors	47	No
WHO	2017	Taeniasis	Experts from academic institutions, industry, and private and public sectors	47	No
WHO	2022	Yaws	Experts from academic institutions	7	No
WHO	2022	Scabies	Experts from academic institutions and public health organizations	6	No
WHO	2022	Scabies	Experts from academic institutions	6	No
FIND, WHO	2020	Diseases in low-resource settings that require point-of-care diagnosis	Experts from academic institutions, industry, and private and public sectors	39	Yes
WHO	2016	Tuberculosis	Experts from academic institutions, industry, and private and public sectors	17	No
FIND	2020	Infectious diseases	Experts from academic institutions, industry, and private and public sectors	51	Yes
WHO, FIND, MSF	2018	Severe febrile illness, HIV, Malaria, TB	No information	52	No

To improve transparency of the TPP development process, we suggest a list of all consulted stakeholders to be included at the end of the TPP document, with their roles in the process and the organizations they represent. In our opinion it is also important to identify and include a broader representation of stakeholders, such as design engineers, and field-based health workers such as Community Health Extension Workers (CHEWs) which are the last link to communities in sub-Saharan Africa (Onasanya et al., 2020; Banda et al., 2021). Inclusion of CHEWs and other lower cadre healthcare staff, in addition to more proxy end users such as researchers and healthcare managers, can support a successful drafting process of TPPs for DHDs that will potentially be used by these CHEWs.

#### 4.1.3 Sustainability

When exploring the TPPs in the database we noticed that sustainability was hardly mentioned in any of the TPPs reviewed, not even in ideal scenarios. In alignment with the SDGs, environmental sustainability in diagnostic device design is an important aspect to consider in the development of TPPs for digital diagnostic devices and should ideally be part of each TPP. Sustainability covers a broad range of parameters including materials used, product lifecycle, and energy requirements and is not limited to product disposal only (Hinrichs-Krapels et al., 2022). Although the emphasis on sustainability can increase the entry threshold for interested companies and can be a barrier to diagnostic device development due to potential limited economic returns on investment, including sustainability in the TPP parameters will bring the topic to the forefront of the product design process, which is the key to designing sustainable products. In addition, including sustainability upfront can foster the co-creation of device development with stakeholders who understand the use context thereby creating a mutually beneficial product.

Our database revealed that components of several reviewed TPPs vary. Taken as a whole, TPPs by WHO and other NGOs showed variation in the level of detail, with more details on the performance and functionality characteristics and less detail on human operational factors such as ease of use and field applicability in low resource settings and ergonomic design. All these can affect contextual fit and sustainability. Other contextual fit and sustainability challenges for DHDs that need to be considered include internet connectivity and limited or no electricity supply in hard-to- reach areas where NTDs are dominant (Onasanya et al., 2023). Considering the use of alternate sources of power supply such as solar technology and use of Bluetooth, Local Area Network (LAN) and external hard drives as alternate sources to sending and keeping diagnostics data should be explored.

Finally, a lack of inclusion of commercial market perspectives can also have an impact on sustainability. Although market perspectives and commercialization may be purposely omitted from the TPP, and there seems to be no market in the NTD space for DHDs, we believe that funding NTDs and improving country ownership of eliminating schistosomiasis and other NTDs will require finding country-based financing mechanisms for digital diagnostics and other diagnostic tools. This can contribute to more sustainable disease control and elimination strategies. We believe there is a need for DHDs and the absence of this perspective in the TPP eliminates its market potential. Including a commercial perspective will require input from health planning officers, health economists and private sector representatives from disease-dominant regions in the TPP drafting process.

### 4.2 Theme 2: interconnectivity and data ownership

While it may appear that TPPs for non-digital diagnostic medical devices may also apply to digital diagnostic medical devices, there are specific concerns for DHPs and DHDs which are not addressed by current TPPs. Ideally, digital diagnostics should be interconnected with other established digital health systems such as health management information systems, logistics management information systems and electronic medical records. These ensure interoperability and reusability across different health program areas, supporting a more robust digital health system. Addressing these at an early stage in the TPPs aligns with the WHO digital health strategy (World Health Organization, 2023a) and enhances a systems approach to the control of NTDs. But interconnectivity also leads to questions concerning data stewardship and the ethical aspects of data sharing policies with third parties such as developers and stakeholders involved in the use of these devices. Consequently, more attention should be paid in TPPs to topics such as data privacy, informed consent, data protection and governance, data storage and data ownership.

## 5 Conclusion

We see great potential for DHPs, DHDs and DODDs that can diagnose schistosomiasis, collate, and can transfer data to digital platforms such as the District Health Information System. DHDs can transform data management systems thereby making healthcare delivery easier and providing decision support for health workers and policymakers. The need for DHDs for NTDs is clear but is mainly driven by the scientific research community, and has not been made actionable by concrete strategies. We found DHDs to be not well embedded in current TPPs, while addressing inclusiveness is important for the further development of DHDs. Our suggestions include the incorporation of end user representatives in the TPP drafting process, more attention to sustainability aspects and addressing interconnectivity gaps and the ethical aspects of data stewardship. Overall, this study highlights the gaps in the current TPPs for diagnostic devices of schistosomiasis, particularly the absence of guidelines for the development of DHPs, surprisingly not even in the ideal scenario. This study also provides an open-access database that others can use to easily search and compare the contents of different diagnostic TPPs for multiple use cases.

## Data availability statement

The datasets generated for this study are available here: https://mud-wheel-f86.notion.site/24e3fa2b55564b0e8ef1cdc4526a3899?v=4a5b9b7a6c004498adf5ec6df308574f”Notiondatabase. Further inquiries can be directed to the corresponding author.

## Author contributions

AO: Writing – original draft, Writing – review & editing. MB: Project administration, Writing – review & editing. LdG: Data curation, Formal Analysis, Investigation, Methodology, Software, Writing – review & editing. JvE: Writing – review & editing. J-CD: Writing – review & editing. LvL: Conceptualization, Funding acquisition, Methodology, Supervision, Validation, Writing – review & editing.
